# Lipopolysaccharide Exposure Induces Maternal Hypozincemia, and Prenatal Zinc Treatment Prevents Autistic-Like Behaviors and Disturbances in the Striatal Dopaminergic and mTOR Systems of Offspring

**DOI:** 10.1371/journal.pone.0134565

**Published:** 2015-07-28

**Authors:** Thiago Berti Kirsten, Gabriela P. Chaves-Kirsten, Suene Bernardes, Cristoforo Scavone, Jorge E. Sarkis, Maria Martha Bernardi, Luciano F. Felicio

**Affiliations:** 1 Department of Pathology, School of Veterinary Medicine, University of Sao Paulo, São Paulo, Brazil; 2 Environmental and Experimental Pathology, Paulista University, Sao Paulo, Brazil; 3 Department of Pharmacology, Institute of Biomedical Science, University of São Paulo, Sao Paulo, Brazil; 4 Institute of Physics, University of São Paulo, Sao Paulo, Brazil; 5 Institute for Energy and Nuclear Research/IPEN, University of São Paulo, Sao Paulo, Brazil; Brock University, CANADA

## Abstract

Autism is characterized by social deficits, repetitive behaviors, and cognitive inflexibility. The risk factors appear to include genetic and environmental conditions, such as prenatal infections and maternal dietary factors. Previous investigations by our group have demonstrated that prenatal exposure to lipopolysaccharide (LPS), which mimics infection by gram-negative bacteria, induces autistic-like behaviors. To understand the causes of autistic-like behaviors, we evaluated maternal serum metal concentrations, which are involved in intrauterine development and infection/inflammation. We identified reduced maternal levels of zinc, magnesium, selenium and manganese after LPS exposure. Because LPS induced maternal hypozincemia, we treated dams with zinc in an attempt to prevent or ease the impairments in the offspring. We evaluated the social and cognitive autistic-like behaviors and brain tissues of the offspring to identify the central mechanism that triggers the development of autism. Prenatal LPS exposure impaired play behaviors and T-maze spontaneous alternations, i.e., it induced autistic-like behaviors. Prenatal LPS also decreased tyrosine hydroxylase levels and increased the levels of mammalian target of rapamycin (mTOR) in the striatum. Thus, striatal dopaminergic impairments may be related to autism. Moreover, excessive signaling through the mTOR pathway has been considered a biomarker of autism, corroborating our rat model of autism. Prenatal zinc treatment prevented these autistic-like behaviors and striatal dopaminergic and mTOR disturbances in the offspring induced by LPS exposure. The present findings revealed a possible relation between maternal hypozincemia during gestation and the onset of autism. Furthermore, prenatal zinc administration appears to have a beneficial effect on the prevention of autism.

## Introduction

Prenatal viral and bacterial infections impair short- and long-term behavior and central nervous system activity in animals [1–3]. Maternal immune activation can also induce neuropsychiatric disorders, including schizophrenia and autism [4–7].

Autism (autism spectrum disorder) is a developmental brain disorder characterized by social deficits, communication abnormalities, repetitive behaviors, and cognitive inflexibility, with a higher prevalence in males [8]. One in every 100 children is diagnosed with autism [9, 10]. The risk factors appear to include genetic and perinatal environmental conditions, such as viral prenatal infections and maternal dietary factors; however, the exact etiology remains unknown [11–13].

Previous investigations by our group have shown that prenatal treatment of rats on gestational day (GD) 9.5 with lipopolysaccharide (LPS; 100 μg/kg, intraperitoneal [i.p.]), an endotoxin that mimics infection with gram-negative bacteria, impaired communication and socialization and induced repetitive/restricted behavior in male offspring. However, the behavior of female offspring was not altered [14, 15]. These results suggest that our model of prenatal LPS exposure induces autism-like behavior in offspring [15]. Moreover, we observed an increase in serum interleukin-1β (IL-1β) levels in adult offspring [16], a finding previously reported in several autistic patients [17–19]. The effects of maternal LPS exposure on the developing fetal brain have been suggested to be mediated by the induction of proinflammatory cytokines within the maternal circulation and placenta [20–22].

To date, no effective treatment exists for autism, and there is no consensus on the type of medication to prescribe [23]. A few drugs have been approved by the U.S. Food and Drug Administration, but these agents have limited efficacy, treat only some of the symptoms, and trigger adverse effects [24]. Therefore, the purpose of the present study was to use our rat model of autism to test a treatment for autism. We selected zinc as the prenatal treatment to prevent or ease the impairments induced by LPS. Cytokines produced after LPS exposure induce metallothionein, which sequesters zinc and induces maternal and fetal hypozincemia [25]. Coyle’s group reported that hypozincemia induced by LPS leads to teratogenesis, and zinc supplementation prevented some of the reproductive and offspring behavioral impairments [25, 26]. Human studies have previously investigated nutritional supplementation with zinc for autism treatment [27, 28]. However, according to Theoharides and colleagues [13], these studies are not representative because they utilized a small number of subjects and did not include appropriate controls. Thus, an evaluation of whether prenatal zinc can reverse the impairments identified in our rat model of autism would be interesting.

First, the maternal serum zinc concentration was measured to evaluate if the autistic-like behaviors induced by our prenatal LPS rat model of autism [15] could be attributed to hypozincemia. Moreover, the concentrations of other metals, such as magnesium (Mg), copper (Cu), selenium (Se) and manganese (Mn), which are involved in pregnancy, intrauterine development, and infection/inflammation [29], were also measured in the dams.

To evaluate if zinc treatment could prevent or ameliorate the autistic-like behaviors in the offspring, we observed play behavior and T-maze spontaneous alternation tests, which are routinely used to measure social deficits, repetitive behaviors, and cognitive inflexibility in autistic-like contexts in rats [15, 30–32]. Finally, we evaluated the brain tissue of the offspring in an attempt to understand the central mechanism that triggers the development of autism, as this mechanism remains unclear. We evaluated the levels of tyrosine hydroxylase (TH) and mammalian target of rapamycin (mTOR) in the striatum and substantia nigra. We have previously demonstrated that prenatal LPS resulted in striatal dopaminergic impairments in adult offspring, including reduced levels of TH, dopamine and metabolites [15, 33]. Furthermore, epidemiological findings and animal models have indicated that excessive signaling through the mTOR pathway leads to an increased risk of autism [34–36].

## Materials and Methods

### Ethics statement

This study was carried out in strict accordance with the recommendations in the Guide for the Care and Use of Laboratory Animals of the National Institutes of Health. The protocol was approved by the Committee on the Ethics of Animal Experiments of the School of Veterinary Medicine, University of São Paulo, Brazil (Permit Number: 2824/2012). All efforts were made to minimize suffering, reduce the number of animals used, and utilize alternatives to in vivo techniques when available. The experiments were performed in accordance with good laboratory practice protocols and quality assurance methods.

### Animals

A total of 47 pregnant Wistar rats between 12 and 14 weeks of age and weighing 222–266 g were used. The rat housing and nutritional conditions, as well as the determination of GD 0 and the handling and care of the dams, were the same as previously described by our group [15]. A total of 17 dams were used to measure the maternal serum metal concentrations. For the offspring studies, 30 dams were allowed to give birth and nurture their offspring under normal conditions. The day of birth was recorded as postnatal day (PND) 1. No handling was performed on PND 1; on PND 2, eight offspring (four males and four females) were randomly selected for the following studies. No cross-fostering procedure was employed. Litters with fewer than eight pups were culled. The pups remained with each dam until weaning (PND 21). On PND 21, the male rat pups were individually housed in polypropylene cages under the same conditions as their parents until PND 30. A maximum of two male rats from each litter were used for offspring evaluations to minimize potential confounding factors associated with the litter [37]. The female offspring were separated for use in other studies.

### LPS exposure

LPS (from Escherichia coli; Sigma, St. Louis, MO; serotype 0127: B8) was dissolved in sterile saline (50 μg/ml LPS in a 0.9% NaCl solution) and administered i.p. to pregnant dams at a dose of 100 μg/kg on GD 9.5. This dose was selected based on our previous findings of maternal sickness behavior and behavioral and brain impairments in offspring [8,10]. The control group, i.e., the SAL group, included pregnant rats that received only sterile saline (0.9% NaCl) according to the same treatment schedule as that of the LPS-treated animals. Each control dam was treated with a 0.2 ml/100 g saline solution.

### Maternal metal concentrations

At 24 h after prenatal exposure to LPS (n = 9) or saline (n = 8), the pregnant rats of the LPS and SAL groups were evaluated to measure the serum concentrations of Zn, Mg, Cu, Se, and Mn by a high-resolution inductively coupled plasma mass spectrometer (HR-ICPMS). A detailed description of this method has been reported by Santos [38] and Anzolin et al. [39]. Trunk blood was collected from the rats on GD 10.5 (2:00 to 4:00 PM). The serum was obtained from the samples by centrifugation. An internal standard solution was obtained with scandium, indium, rhodium, and nitric acid. The calibration curve was obtained via the addition of various concentrations of the element of interest in a serum sample. The quality assurance methods included blanks, duplicates and analysis of the certified reference material seronorm trace element serum levels every 10 samples.

### Zn treatment

For the offspring studies, three groups were investigated. (1) A SAL+SAL group (also referred to as the control group) consisted of pregnant rats that received sterile saline on GD 9.5 (0.2 ml/100 g, i.p.) and an additional saline injection after 1 h (0.2 ml/100 g, subcutaneous in the nape of the neck [s.c.]). Saline was used as the vehicle for both LPS and zinc. (2) The LPS+SAL group consisted of pregnant rats that received LPS on GD 9.5 (100 μg/kg, i.p.) and a saline injection after 1 h (0.2 ml/100 g, s.c.). (3) The LPS+Zn group consisted of pregnant rats that received LPS on GD 9.5 (100 μg/kg, i.p.) and a zinc injection (zinc sulfate heptahydrate, ZnSO_4_, Sigma, St. Louis, MO, USA, cat. no. Z0635; 2 mg/kg in 0.9% saline, s.c.) after 1 h. The zinc dosage, route and interval of administration were based on the findings of Coyle’s group [40]. A s.c. zinc injection induces an immediate and consistently reproducible increase in plasma zinc that peaks at a level that is four- to fivefold higher than baseline 2 h after injection and returns to normal by 12 h [41]. No evidence has been reported that these plasma zinc levels have a detrimental effect on pregnancy outcome [26]. The recovery of normal zinc levels 12 h after s.c. zinc injection coincides with the period of increased cytokine levels after LPS exposure [42, 43]. The zinc solution was always prepared on the day of administration. All of the experiments were performed between 9:30 and 11:00 AM to minimize the effects of circadian rhythms. Testing between groups was intermixed.

### T-maze

Repetitive/restricted behavior and cognitive inflexibility are some of the most typical symptoms of autism [8]. These behaviors were evaluated using the T-maze spontaneous alternation test, based on Timofeeva et al.’s studies [44, 45]. Although T-maze is a behavioral test typically used for several proposes, such as investigation of spatial working memory, learning and memory studies, and anxiety [46, 47], it is also used for autistic-like behavior analyzes [48]. On PND 29, two offspring per litter for each group (SAL+SAL, LPS+SAL, and LPS+Zn, n = 10 per group) were evaluated in the T-maze. The T-maze was composed of waterproof black-painted wood with three arms in a T-shape (90°). The arms were 10-cm wide, and the walls were 30-cm high. One of the arms had a 20-cm long starting compartment separated from the rest of the arm by a removable wall. The remainder of the stem of the maze was 45-cm long. The two other arms (left and right) were the free choice arms and were 40-cm long. The testing room was small and dimly lit. The rats were placed in the start area for 10 sec. At this point, the barrier was raised, and the rat was permitted to explore the maze for up to 30 sec. Once the rat entered into one of the free choice arms, the barrier was inserted to block the animal in that arm for 30 sec. Thereafter, the rat was repositioned in the start area, initiating another session. If the rat did not enter any of the free choice arms after 30 sec, the rat was replaced in the start area, and the session was restarted. Five sessions were performed for each rat. For each session, the first choice of the rat in the free choice arms was evaluated, i.e., whether the rat first entered the left or right arms. The parameter analyzed was the percentage (%) of alternation between the left and right arms, which was always assessed in relation to the arm visited in the previous session. This model is based on the natural proclivity of rats to alternate between the visited goal-arms in each trial over a series of successive trials [46]. Thus, a higher percentage of alternation between the arms was considered normal rat behavior, whereas less alternation indicated cognitive inflexibility and repetitive behavior. For statistical analysis, these data were transformed into scores: 0 = no alternations, i.e., repeatedly visiting the same arm for all five sessions; 1 = one alternation; 2 = two alternations; 3 = three alternations; and 4 = four alternations, i.e., always alternating between the visited arms for all five sessions. The apparatus was washed with a 5% ethanol/water solution prior to the first session to eliminate the potential biasing effects of odor cues left by the previous rat.

### Play behavior

Impaired social interaction and play behavior are some of the most typical symptoms of autism [8]. These behaviors were evaluated using the play behavior test, based on our previous studies [14, 15]. The same two offspring per litter for each group (SAL+SAL, LPS+SAL, and LPS+Zn, n = 10 per group) observed in the T-maze were also evaluated for play behavior. Briefly, on PND 21, the rat pups were individually housed in polypropylene cages (38 x 32 x 16 cm) under the same conditions as their parents until PND 30. The rationale behind the social isolation was to increase the motivation to initiate play behavior [49]. Play behavior was evaluated on PND 30 because this behavior has been shown to peak during this time [30]. For the evaluation, each isolated rat in the three groups was paired with a naïve male rat (i.e., without any treatment) that has previously been housed in a group environment. The weight difference of the two rats (isolated and naïve-grouped) was up to 10 g. Each naïve rat was only used for one pairing. The testing room was small and dimly lit, with a video camera mounted near the ceiling to record behavior. A 5 min period was allowed for the animals to adapt to the testing room prior to matching. The naïve-grouped rat was always placed into the cage of the isolated rat, where the test was conducted; therefore, the isolates are also referred to as the residents, and the grouped rats are referred to as the intruders. Their behaviors were recorded for 10 min in the testing room isolated from the experimenter. The following parameters were measured only for the isolated rats: pinning frequency (the frequency of play behavior, i.e., the number of times the resident rat laid on its back and showed his belly to the intruder, which mounted the resident from above to complete the social interaction), darting frequency (the number of times the resident moved rapidly towards, in parallel, or away from the intruder), the frequency of crawls over/under the intruder, the duration of time (sec) spent following the intruder, the time (sec) spent sniffing the intruder, and the rearing frequency (the number of times the resident rat stood on its hind legs without interacting with the intruder). Pinning is considered social play, darts and crawls over/under are considered play solicitations, following and sniffing the intruder are considered social investigations, and rearing is considered a non-social exploratory behavior [30].

### TH and mTOR expression

The TH and mTOR protein levels in the striatum and substantia nigra were quantitatively analyzed via western blot as previously described [50, 51]. At 4 to 5 h after the play behavior test, i.e., between 1:00 and 3:00 PM, the brain was collected after decapitation, and the striatum and substantia nigra were rapidly dissected and homogenized in an extraction buffer. These animals were at PND 30, and there were two offspring per litter for each group: SAL+SAL, LPS+SAL, and LPS+Zn (n = 6–7 per group). This method has previously been described by Rong and Baudry [52]. Briefly, the homogenates were subjected to centrifugation, and the protein concentration of the supernatant was determined using a protein assay (Bio-Rad, Hercules, CA). The samples from the homogenate were separated on an acrylamide gel and electrotransferred to nitrocellulose membranes using a Trans-Blot cell system (Bio-Rad). The nitrocellulose membranes were then blocked and incubated with primary monoclonal antibodies: mouse anti-TH (Chemicon, Temecula, CA) and rabbit anti-mTOR (Cell Signaling Technology, Danvers, MA). In all experiments, an anti-Beta-actin antibody (Sigma) was used as a loading control. The bound antibodies were visualized using a chemiluminescence kit (ECL Kit; Amersham Biosciences, Little Chalfont, Buckinghamshire, United Kingdom). Finally, the blots were densitometrically analyzed in ImageJ (NIH/USA). Because there were no changes in the expression levels of Beta-actin, the optical densities of the TH and mTOR bands were normalized to the corresponding Beta-actin bands in each experiment. The digital images were collected and processed using Adobe Photoshop 7.0.1 software (Adobe Systems, Inc.). For all image acquisitions, the bands were contained in the same gel, and the most representative bands were selected.

### Statistical analysis

Homogeneity was verified using an F test or Bartlett’s test. Normality was verified using the Kolmogorov-Smirnov test. Student’s *t*-tests (unpaired, two-tailed) were used to compare the parametric data between the two groups. One-way analysis of variance (ANOVA) followed by Bonferroni’s multiple comparison test was used to compare the parametric data among the three groups. For the T-maze score analysis, a Kruskal-Wallis test was used, followed by a Dunn’s test. The results are expressed as the mean ± SEM or the median (minimum and maximum). In all cases, the results were considered significant at *p* < 0.05.

## Results

Maternal LPS exposure decreased the serum Zn, Mg, Se and Mn concentrations compared with the control dam data 24 h after the treatment (Table 1, *p* < 0.05 in all cases). The Cu concentration was not significantly different between the LPS and control groups. Because it was observed that maternal LPS exposure induced hypozincemia, the dams were treated with zinc in an attempt to prevent or ameliorate the impairments in the offspring.

**Table 1:** Maternal metal concentrations. The effects of LPS (100 μg/kg) exposure at gestational day 9.5 in rats on maternal serum metal concentrations (μg.kg^-1^) measured 24 h after treatment. SAL, prenatal saline injection (n = 8); LPS, prenatal LPS injection (n = 9). Data are expressed as the mean ± SEM.

Element	SAL group	LPS group	*P*	t	df
Zinc	742.32 ± 22.22	676.31 ± 23.94 *	0.0317	2.0040	15
Magnesium	21528.17 ± 296.05	19898.69 ± 587.65 *	0.0310	2.3803	15
Copper	1394.12 ± 72.92	1313.92 ± 45.08	0.3526	0.9592	15
Selenium	359.73 ± 10.18	326.20 ± 8.07 *	0.0197	2.6097	15
Manganese	5.96 ± 1.09	3.19 ± 0.69 *	0.0447	2.1902	15

*p < 0.05 compared with the SAL group (Student’s t-test).

**Fig 1:**
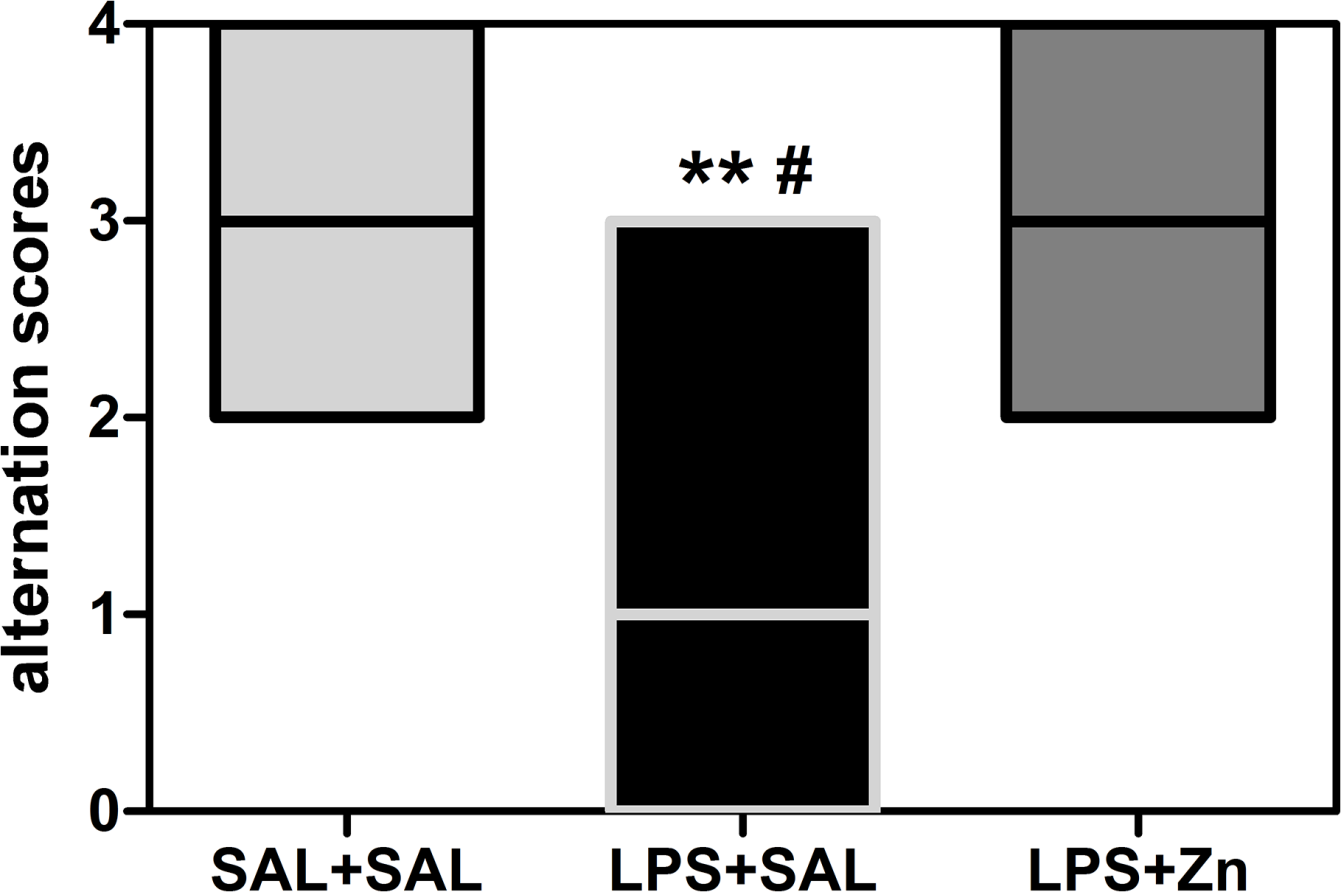
T-maze spontaneous alternation of the offspring. The effects of prenatal LPS (100 μg/kg) and zinc (ZnSO_4_; 2 mg/kg) exposure at gestational day 9.5 on T-maze spontaneous alternation in young male rat offspring (PND 29). Five-session scores: 0, no alternations; 1, one alternation; 2, two alternations; 3, three alternations; and 4, four alternations. SAL+SAL, prenatal saline injection and another saline injection 1 h later; LPS+SAL, prenatal LPS injection and a saline injection 1 h later; LPS+Zn, prenatal LPS injection and a zinc injection 1 h later (*n* = 10 rats/group). **p < 0.01 compared with the SAL+SAL group; ^#^ *p* < 0.05 compared with the LPS+Zn group (Kruskal-Wallis test followed by Dunn’s test). The data are expressed as the median (minimum and maximum).

The performance in the T-maze spontaneous alternation task was different between groups (KW = 10.95, *p* = 0.0042, Fig 1). Prenatal LPS exposure decreased T-maze spontaneous alternation in the offspring compared with that in the control group (*p* < 0.01). Post-treatment with zinc increased spontaneous alternation in the rats prenatally exposed to LPS (LPS+Zn group *vs*. LPS+SAL group, *p* < 0.05) to the same levels as those in the control group. Thus, prenatal LPS induced repetitive/restricted behavior and cognitive inflexibility, and zinc treatment prevented these impairments.

**Fig 2:**
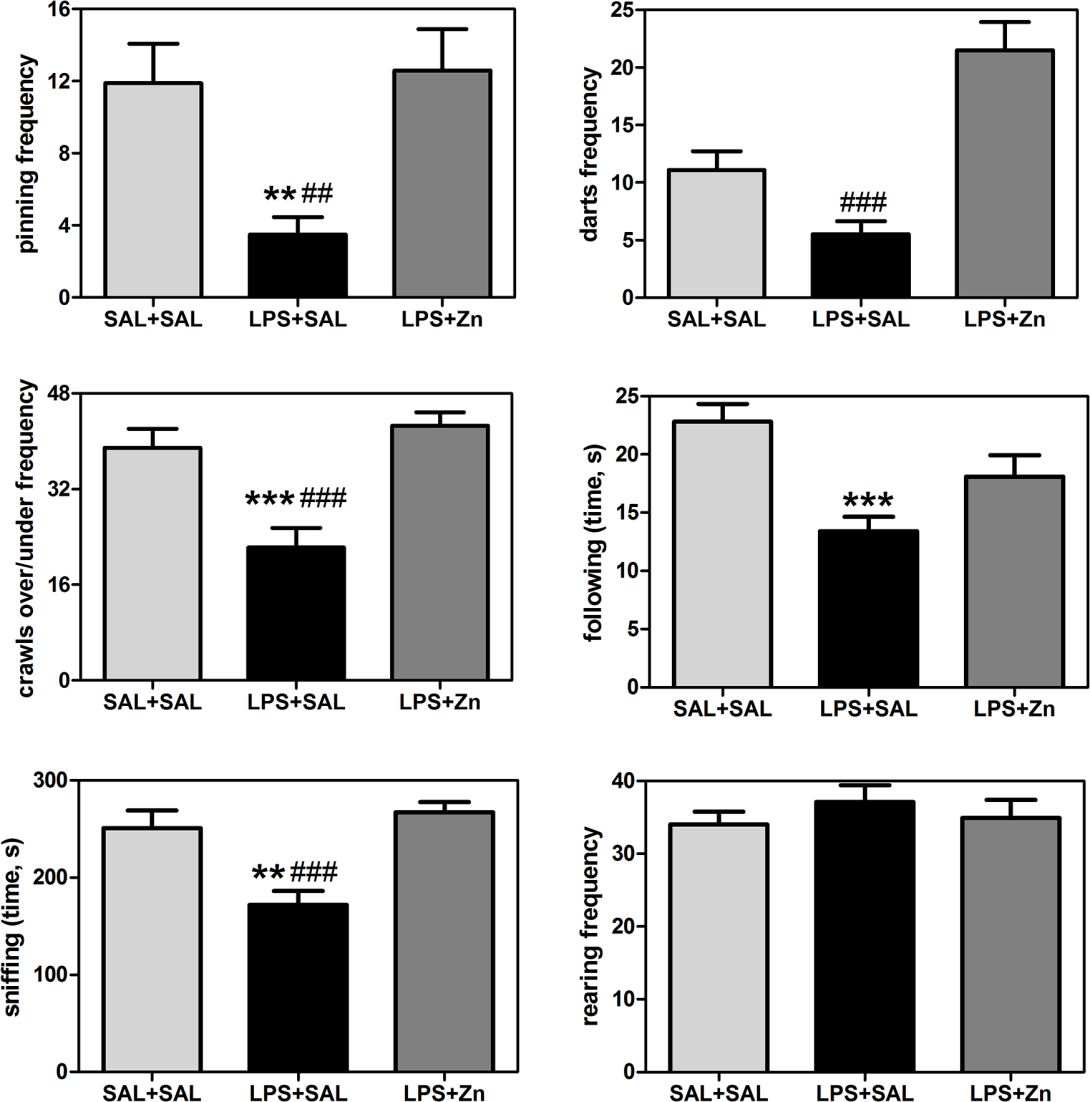
Play behaviors in the offspring. The effects of prenatal LPS (100 μg/kg) and zinc (ZnSO_4_; 2 mg/kg) exposure at gestational day 9.5 on play behaviors in young male rat offspring (PND 30). SAL+SAL, prenatal saline injection and another saline injection 1 h later; LPS+SAL, prenatal LPS injection and a saline injection 1 h later; LPS+Zn, prenatal LPS injection and a zinc injection 1 h later (*n* = 10 rats/group). **p < 0.01 and ***p < 0.0001 compared with the SAL+SAL group; ^#^ *p* < 0.01 and ^###^ *p* < 0.0001 compared with the LPS+Zn group (one-way ANOVA followed by the Bonferroni test). The data are expressed as the mean ± SEM.

The play behavior performance was different between groups (Fig 2): pinning (F(2/27) = 7.04, *p* = 0.0035), darts (F(2/27) = 19.87, *p* < 0.0001), crawls over/under (F(2/27) = 13.46, *p* < 0.0001), following (F(2/27) = 9.13, *p* = 0.0009), and sniffing (F(2/27) = 11.71, *p* = 0.0002), but not for rearing (F(2/27) = 0.52, *p* = 0.6011). Prenatal LPS exposure also impaired play behavior in the offspring, i.e., the rats in the LPS+SAL group exhibited decreased pinning (*p* < 0.01), crawls over/under (*p* < 0.0001), following (*p* < 0.0001), and sniffing (*p* < 0.01) compared with the behavior exhibited by the control group. Post-treatment with zinc increased play behavior in the rats prenatally exposed to LPS, i.e., the rats in the LPS+Zn group exhibited increased pinning (*p* < 0.01), darts (*p* < 0.0001), crawls over/under (*p* < 0.0001), and sniffing (*p* < 0.0001) compared with the LPS+SAL group, reaching the same levels exhibited by the control group. The rearing frequency did not vary significantly among the three groups, which indicates there were no changes in non-social exploratory activity. Thus, prenatal LPS impaired social play, play solicitations, and social investigations, and zinc treatment prevented these impairments.

**Fig 3:**
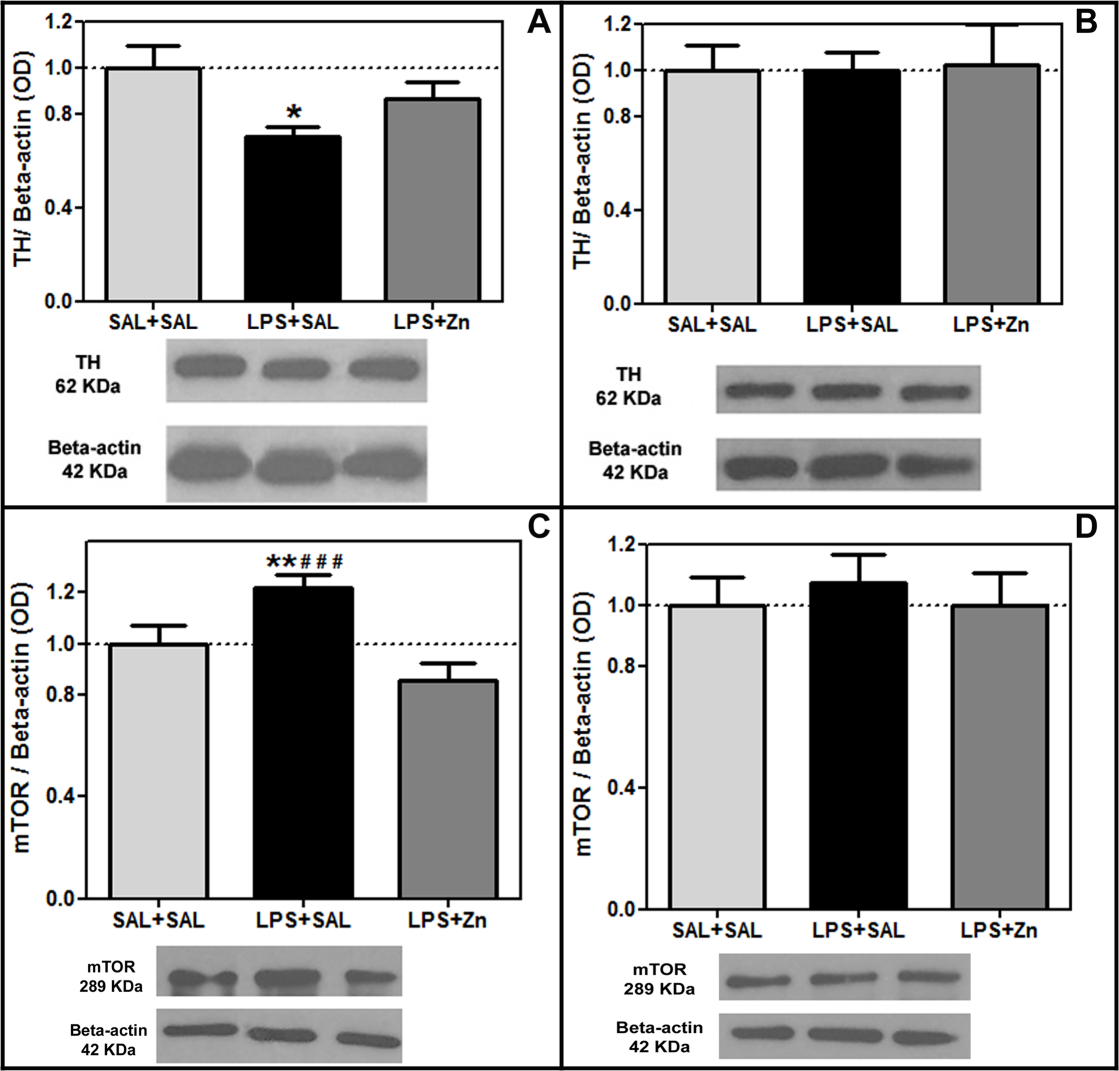
TH and mTOR expression in the offspring. The effects of prenatal LPS (100 μg/kg) and zinc (ZnSO_4_; 2 mg/kg) exposure at gestational day 9.5 on the TH and mTOR levels in the striatum and substantia nigra (normalized optical density/Beta-actin ratio, western blotting) in young male rat offspring (PND 30). (A) striatal TH levels; (B) substantia nigra TH levels; (C) striatal mTOR levels; (D) substantia nigra mTOR levels. SAL+SAL, prenatal saline injection and another saline injection 1 h later; LPS+SAL, prenatal LPS injection and a saline injection 1 h later; LPS+Zn, prenatal LPS injection and a zinc injection 1 h later (*n* = 6–7 rats/group). *p < 0.05 and **p < 0.01 compared with the SAL+SAL group; ^###^ *p* < 0.0001 compared with the LPS+Zn group (one-way ANOVA followed by the Bonferroni test). The data are expressed as the mean ± SEM.

The TH protein levels were different between groups in the striatum (F(2/18) = 4.27, *p* = 0.0304, Fig 3A). The TH protein levels were reduced by prenatal LPS exposure in the striatum of the offspring compared with those in the control group (*p* < 0.05). Post-treatment with zinc was unable to increase the striatal TH levels in the rats prenatally exposed to LPS (LPS+Zn group *vs*. LPS+SAL group, *p* > 0.05). However, the striatal TH levels of the control and LPS+Zn groups were not significantly different, which indicates a potential dopaminergic preventive effect induced by zinc treatment.

The mTOR protein levels were different between groups in the striatum (F(2/21) = 11.13, *p* = 0.0005, Fig 3C). The mTOR protein levels were increased by prenatal LPS exposure in the striatum of the offspring compared with that of the control group (*p* < 0.01). Post-treatment with zinc decreased the striatal mTOR levels in the rats prenatally exposed to LPS (LPS+Zn group *vs*. LPS+SAL group, *p* > 0.0001) to the same levels as those of the control group. Thus, prenatal LPS increased the striatal mTOR levels, and zinc treatment prevented this hyperactivity. The TH and mTOR protein levels in the substantia nigra did not vary significantly among the three groups (F(2/16) = 0.01, *p* = 0.9897, Fig 3B, and F(2/18) = 0.20, *p* = 0.8174, Fig 3D, respectively).

## Discussion

Prenatal infection/inflammation on GD 9.5 falls within a critical period for brain organogenesis. Infections associated with immunological events during the early/middle fetal stages (e.g., GD 8–10 in rats and mice) may have a stronger impact on neurodevelopment compared with infections that occur during late-stage pregnancy. Maternal immune activation during early/middle pregnancy may interfere with cell proliferation, differentiation, migration, target selection, and synapse maturation, which may later lead to multiple brain and behavioral abnormalities in adulthood [53–56]. Previous data from our group have corroborated that GD 9.5 is a critical period. We have demonstrated that prenatal exposure to LPS on GD 9.5 in rats induced short- and long-term reproductive, behavioral, and neuroimmune impairments in the offspring [14–16, 33, 57–59].

In this study, we have shown that the short- and long-term reproductive, behavioral, and neuroimmune impairments in the offspring previously [14–16, 33, 57–59] and currently identified by our group appear to be associated with maternal hypozincemia induced by LPS exposure. We demonstrated that prenatal LPS exacerbates proinflammatory cytokine production in the offspring, resulting in increased serum IL-1β levels [16]. These proinflammatory cytokines induce metallothionein, which sequesters zinc and induces maternal and fetal hypozincemia [25]. It is important to note that, as reported by Coyle and colleagues, no specific storage sites of zinc exist in the body, therefore zinc concentrations must be maintained to ensure an adequate supply to the fetus for normal fetal development [25]. Thus, there are no specific zinc reserves to compensate for a temporary deficiency in maternal zinc. We suggest that if we presently showed that LPS induced maternal hypozincemia, then we can speculate that there was also a fetal hypozincemia. However, further studies should be conducted to confirm if LPS reduces zinc also in fetus and whether zinc supplementation normalized zinc in dam and fetuses.

Zinc is one of the most important trace elements in mammals, and it is required for many physiological processes, such as cell proliferation and differentiation, growth and development, and the regulation of enzymatic activity [60]. Zinc is also known to play a regulatory role in the nervous and immune systems, and it participates in innate and adaptive immunity [61, 62]. Particularly during intrauterine development, zinc deficits can induce reproductive impairments, such as low birth weight and preterm delivery [63]. Thus, zinc supplementation during pregnancy is frequently recommended by health professionals and serves to reduce reproductive impairments both in the mother and fetus; zinc is well tolerated and accepted by patients, without inducing adverse effects [64, 65].

Prenatal exposure to LPS also reduced the levels of magnesium, selenium and manganese. In this regard, it is known that reduced levels of minerals, trace elements and vitamins can induce significant reproductive losses. The elements zinc, magnesium, selenium, manganese, copper, and chromium are all involved in these processes [29]. Magnesium acts in the metabolism of ADP-ATP during the activity of neurons and muscle cells; lower levels are involved in preterm birth, preeclampsia and eclampsia, which leads to maternal and fetal/neonatal morbidity and mortality [66, 67]. Selenium requirements are increased during pregnancy due to transport for the growing fetus [29], and selenium deficiency may cause a predisposition toward demyelization and oligodendrocyte injuries (which have been postulated to induce mental retardation [68]), neural tube injuries [69], and spontaneous abortions [70]. Manganese is an essential mineral nutrient required for proper fetal development and other metabolism processes; maternal blood manganese deficiency is associated with delayed intrauterine fetal growth and reduced birth weight [71]. Therefore, maternal zinc, magnesium, selenium, and manganese deficiencies can induce persistent reproductive and behavioral impairments in offspring. In the present study, we treated dams with zinc to prevent this hypozincemia. Our findings indicate that prenatal zinc treatment was successful. We suggest that future studies should examine supplementation with magnesium, selenium, and manganese during pregnancy stricken by infectious/inflammatory processes.

Our findings of plasma hypozincemia after LPS exposure are significant, though marginal (~10%). Comparing with Coyle et al studies, they observed a massive decrease of zinc levels after LPS exposure in mice [26, 40]. However, the most significant decrease of zinc levels in the Coyle and colleagues studies were between 6 and 16 h, with a marginal decrease 24h after the LPS exposure. Considering that we used the same dose and via (2 mg/kg, s.c. in the nape of the neck), we believe that the most significant decrease of zinc levels in the present study were between 6 and 16 h. Thus, although literature reports studies describing zinc concentrations in fetuses and time course in reduction in zinc after LPS in non-pregnant dams [26, 72], those studies involve mice and such evidence in rats is required. On the other hand the same group reports that the reduction in pregnant mice is much less (38%), and does not provide the data for fetuses [40]. There is also evidence that LPS decreases plasma zinc in dams, and that there are observable effects on fetal outcome that can be prevented by zinc supplementation [25, 40, 72]. Nevertheless, there is limited understanding of the mechanisms, and further studies, such as zinc in fetuses and in dams after treatment, should be performed.

Prenatal LPS exposure decreased T-maze spontaneous alternation in the offspring, i.e., prenatal LPS exposure induced repetitive/restricted behavior and cognitive inflexibility. Moreover, prenatal LPS impaired socialization in the juvenile offspring, i.e., it reduced the amount of social play, play solicitation, and social investigation during the play behavior test. Therefore, prenatal LPS exposure induced the most typical symptoms of autistic-like behaviors in the offspring. These data are in accordance with our previous studies of an autism rat model [14, 15]. Incidentally, epidemiological studies and experimental animal models have indicated an association between maternal immune activation/infection during pregnancy and an increased risk of central nervous system disorders in offspring, including schizophrenia, autism, and cerebral palsy [1, 4–6]. For example, a well-established animal model is based on prenatal treatment with the viral-mimic inflammatory agent polyriboinosinic-polyribocytidilic acid (poly[I:C]), a synthetic analog of double-stranded RNA. Prenatal poly(I:C) exposure in mice is a powerful experimental tool to induce and investigate the distinct brain and behavioral abnormalities associated with schizophrenia, with early/middle (GD 9) and late (GD 17) exposure being relevant to the positive and negative cognitive symptoms, respectively [54, 73–75].

Prenatal/perinatal exposure to numerous pathogens, including rubella, measles, and cytomegalovirus, has been implicated in the etiology of autism, suggesting that the infection-associated risk of autism might not be pathogen specific [5]. This hypothesis is supported by a hospital study suggesting that maternal exposure to various viral or bacterial infections significantly increased the risk of autism-spectrum disorders in children, and this effect appeared to be unrelated to hospitalization per se [4]. Thus, acute fetal neuroinflammation, together with its effects on early neurodevelopmental processes, may facilitate development of the psychopathological and neuropathological phenotypes of autism [5].

The social interaction/ play behavior impairment found after prenatal LPS exposure was induced after social isolation of rats, i.e., they were individually housed in the previous days of the test. The rationale behind the social isolation was to increase the motivation to initiate play behavior [49]. This procedure helps to reveal social changes after the treatments, and is a standard method also used for rat models of autism [30]. Particularly in our model of prenatal LPS exposure, we previously showed that without the social isolation of the rat, the impairments were not revealed [14]. Thus, it is important to proceed with social isolation before the play behavior test.

Social interactions could also be affected by anxiety. However, we have already investigated anxiety levels in our model of prenatal LPS exposure with a specific behavioral test of anxiety: the elevated plus maze test [14]. The elevated plus maze is currently one of the most popular tests for anxiety [76]. We showed that prenatal LPS exposure did not influenced anxiety parameters, i.e., did not induced anxiolytic nor anxiogenic effects.

Prenatal zinc administration prevented cognitive (T-maze) and social (play behavior) impairments induced by LPS exposure, restoring these rat behaviors to the same levels exhibited by the controls. Therefore, prenatal zinc prevented autistic-like behaviors in rats. According to Caulfield et al. [77], prenatal zinc supplementation without inflammatory processes does not influence cognitive skills, social skills or behavioral development. Thus, it appears that prenatal zinc induces behavioral changes only after an inflammatory process (e.g., induction by LPS).

We believe that these results demonstrating that prenatal zinc treatment prevents autistic-like behaviors may generate significant interest with potential clinical extrapolation. First, our rationale is primarily based on the lack of effective treatments for autism to date [23]; the current drugs have limited efficacy and trigger adverse effects [24]. Second, it is well known that zinc supplementation is recommended during pregnancy because it reduces reproductive impairments in both the mother and fetus and is well tolerated and accepted by patients, without inducing adverse effects [64, 65]. Thus, regarding the extrapolation to humans, we suggest that when the first signs of sickness behavior associated with an infection are perceived in a pregnant woman, such as those induced by LPS, zinc could be administered to prevent the development of autism in newborns.

Finally, we evaluated the brain tissue of the offspring to investigate central mechanisms that may underlie the behavioral differences observed. Prenatal LPS exposure decreased striatal TH levels in the juvenile offspring. These data are in accordance with those of our previous studies, which identified other striatal dopaminergic impairments in adult offspring, such as reduced levels of dopamine and its metabolites [15, 33]. In this regard, LPS is a potent inducer of IL-1β and tumor necrosis factor-alpha, which degenerate and kill dopaminergic neurons [78, 79].

There are several neurological hypotheses for autism, the most widespread of which is hyperserotonemia [80]. However, serotonin levels have been shown to be unaffected by prenatal LPS exposure [81]. A dopaminergic hypothesis also exists, suggesting that hyperactivation of the dopaminergic system leads to autism [82]. The fact that autism is defined as having a ‘‘broader phenotype” [83] may explain the varied neurological manifestations that have been reported. Considering the striatal dopaminergic impairment together with the autistic-like behaviors induced by prenatal LPS exposure, we have suggested hypoactivity of the dopaminergic system as a potential mechanism underlying autism-spectrum disorder.

Post-treatment with zinc revealed a possible dopaminergic preventive effect because striatal TH levels in the control and LPS+Zn groups were not significantly different. In fact, prenatal zinc oxide exposure increases dopamine and metabolite levels in the hippocampus and prefrontal cortex of mice [84].

Epidemiological findings and animal models have indicated that excessive signaling through the mTOR pathway increases the risk of autism [34]. The mTOR pathway is currently under investigation for the treatment of autism [34–36]. For example, it has been postulated that constitutively increased mTOR signaling could be offset by rapamycin, an mTOR inhibitor [85]. Because of the well-established relation between autism and mTOR, we evaluated the brain mTOR levels in our autism rat model. We identified increased striatal mTOR levels after prenatal LPS exposure. Together with our data on impaired communication and socialization, repetitive/restricted behaviors, and increased serum IL-1β levels [14–16], we concluded that our model of LPS exposure in prenatal rats on GD 9.5 is a robust rat model of autism.

Post-treatment with zinc following prenatal exposure to LPS decreased the striatal mTOR to levels similar to those of the control group, i.e., prenatal zinc prevented the hyperactivity of the mTOR pathway. To this end, extracellular zinc activates p70S6 kinase, a downstream component of the mTOR pathway [86]. Incidentally, zinc has been postulated as a stimulant of embryonic stem cell proliferation through the mTOR signal pathway [87]. Thus, a role for maternal infection-induced zinc deficiency in autism of the offspring appears likely.

In conclusion, prenatal LPS exposure on GD 9.5 induced social deficits, repetitive behaviors, and cognitive inflexibility in juvenile rat offspring. In other words, our rat model induced autistic-like behaviors. The brains of these juvenile rat offspring were also affected by prenatal LPS and exhibited decreased TH levels and increased mTOR levels in the striatum. Thus, striatal dopaminergic impairments may be associated with autism. Moreover, excessive signaling through the mTOR pathway has been considered a biomarker of autism [34], which corroborates our rat model of autism. These behavioral and brain impairments were associated with maternal hypozincemia during gestation induced by LPS exposure. Prenatal zinc treatment prevented the autistic-like behaviors and striatal dopaminergic and mTOR disturbances in the offspring induced by LPS exposure. The present findings indicate a potential relation between maternal hypozincemia during gestation and impairments in cognition and social (play) behavior. Furthermore, prenatal zinc administration appears to have a beneficial effect on the prevention of these behavioral impairments, which are related to experimental autism. The present findings may contribute to a better understanding and prevention/treatment of autism and associated diseases.
